# Snacking for a Cause: Nutritional Insufficiencies and Excesses of U.S. Children, a Critical Review of Food Consumption Patterns and Macronutrient and Micronutrient Intake of U.S. Children

**DOI:** 10.3390/nu6114750

**Published:** 2014-10-30

**Authors:** Julie Hess, Joanne Slavin

**Affiliations:** Department of Food Science and Nutrition, University of Minnesota, 1334 Eckles Ave, St. Paul, MN 55108, USA; E-Mail: jmhess@umn.edu

**Keywords:** yogurt, what we eat in America, WWEIA, national health and nutrition examination survey, NHANES 2009–2010

## Abstract

The objective of this review was to identify dietary insufficiencies and excesses in children aged two to 11 in the United States (U.S.) and eating habits that merit concern in terms of nutrient and energy density to improve overall diet quality. Data from the What We Eat in America (WWEIA) tables from the National Health and Nutrition Examination Survey (NHANES) were examined as well as survey data from the School Nutrition Dietary Assessment Study (SNDA). Analysis of survey data revealed that children consume insufficient Vitamin D, calcium, and potassium and excess energy, carbohydrates, and sodium. Dietary modifications are necessary to prevent serious deficiencies and the development of chronic illness. Snacking has steadily increased in this population since the 1970s, and snacks provide necessary nutrients. However, carbohydrates and added sugars tend to be over-consumed at snacking occasions. Replacement of current snack choices with nutrient-dense foods could lower the risks of nutrient deficiencies and help lower excess nutrient consumption. Increased consumption of low sugar dairy foods, especially yogurt, at snack times could increase intake of important micronutrients without contributing to dietary excesses.

## 1. Introduction

The United States (U.S.) has the third highest healthcare expenditure in the world [1]. In 2010, over 17% of the U.S. gross domestic product was spent on healthcare [2]. Preventative care measures, including dietary improvements, could help reduce these costs over time, especially through encouraging health-promoting practices for children. Over 30% of U.S. children and adolescents are overweight or obese [3], which increases their risk of becoming overweight or obese adults [4]. However, even though they consume excess energy, American children consume insufficient nutrients.

The National Health and Nutrition Examination Survey (NHANES) evaluates the health and diet of the U.S. population. NHANES data show that children aged two to 11 have low overall intakes of fiber, Vitamin D, calcium, and potassium, but an excess consumption of added sugars and refined carbohydrates, in addition to energy. Too little Vitamin D, calcium, and potassium can lead to a wide range of health problems later in life, including osteoporosis, hyperparathyroidism [5] and hypertension [6]. Energy and refined carbohydrate-laden diets can also lead to an increased susceptibility to becoming overweight or obese and to developing cardiovascular disease [7,8]. Furthermore, nutrient-poor diets consumed during childhood can establish a lifetime pattern of poor eating habits [9].

To improve the health of the U.S. population, it is vital to address the nutrition of its children and promote positive change in children’s eating choices. The 2010 Dietary Guidelines for Americans (DGA) provide the official nutrition recommendations for Americans two years of age and older and include information on how to structure a health-promoting diet. The 2010 DGA identify potassium, dietary fiber, calcium, and Vitamin D as “nutrients of concern” across the population [9]. The intake levels of these nutrients are low enough across the population to be a concern for public health [9]; data from the 2009–2010 NHANES also show low intake levels of these four nutrients among children aged two to 11 [10] (the only exception to the low levels of nutrients was calcium consumption for two to five year olds, which slightly exceeded the recommended—Dietary Reference Intake). In conjunction with the “nutrients of concern,” the 2010 DGA include a list of foods Americans should consume more frequently [9]. This list includes a variety of fruits and vegetables, whole grains, low fat milk and milk products, seafood and other lean protein options, and oils [9]. Vegetables, fruits, dairy products, and whole grains are all mentioned as good food sources for the nutrients of concern [9]. In addition to nutrients of concern and foods to eat more of, the 2010 DGA list food components to reduce, which include sodium, saturated fat, trans fat, cholesterol, added sugar, solid fat, and refined grains [9].

MyPlate, the visual representation of the 2010 DGA, is another source for U.S. dietary recommendations. The MyPlate graphic is a plate divided into sections by food group (vegetables, fruits, grains, protein foods, and dairy) [11]. The size of each section on the plate depicts how much of each food group should be consumed daily. For example, vegetables and grains are the largest two sections on the plate, reflecting recommendations that Americans consume more vegetables and more whole grains [11]. MyPlate also encourages Americans to be physically active and to avoid dietary sources of solid fats, added sugars, and excess sodium [11].

In addition to providing information regarding the overall daily intakes of each food group, NHANES data also include information on when children tend to consume different nutrients. Encouraging changes to the nutrient profile of different eating occasions is one possible method of improving children’s eating habits.

## 2. Data Sources

The dietary data used for this analysis come primarily from the What We Eat in America (WWEIA) tables of the 2009–2010 NHANES and School Nutrition Dietary Assessment Study (SNDA) data. NHANES is an annual survey of American children and adults conducted by the Centers for Disease Control and Prevention, the United States Department of Agriculture (USDA), and the National Center for Health Statistics to study the health of the national population, including its dietary habits. WWEIA data are cross-sectional data based on two days of 24-h dietary recall. The NHANES data used originates from the WWEIA tables available on the USDA website and from published scientific articles. Publications used include Centers for Disease Control and Prevention and USDA publications as well as articles found via keyword searches on government institution websites and scientific databases such as PubMed and ScienceDirect. NHANES 2009–2010 data was the source of information for children’s breakfast, lunch, snack, and dinner consumption habits.

Information on children’s lunches also comes from the SNDA, which evaluates the nutritional quality of meals offered to school-aged children and adolescents through the National School Lunch Program (NSLP) and the impact of this program on children’s health. The USDA’s Food and Nutrition Service contracted with Mathematica Policy Research to conduct the SNDA-IV [12]. The SNDA has been administered periodically since 1991 [13]. Data from SNDA-III (2004–2005) and SNDA-IV (2009–2010) were used in this publication. SNDA data were used in conjunction with NHANES data for lunch consumption information because many elementary school students (63%) participate in the NSLP [13]. The NSLP follows recommendations outlined in the 1995 School Meals Initiative for Healthy Children (SMI). SMI recommendations are based on Recommended Daily Allowances and the DGA [13]. SMI identifies “target nutrients” for school lunches, including protein, vitamin A, vitamin C, calcium, and iron [13]. The SNDA distinguishes between foods “offered” *versus* foods “served” at school lunches. Foods offered accounts for all options available for students to choose. Foods served includes the foods that students select or are given for lunch.

Food consumption and macro- and micronutrient intake of children aged two to 11 from WWEIA tables and the SNDA-IV were compared with existing dietary recommendations to determine inconsistencies between recommendation and practice. Macronutrients studied include energy, carbohydrates, protein, sugar, fiber, and fat. Micronutrients studied include calcium, vitamin D, vitamin B12, magnesium, sodium, and potassium. NHANES data, SNDA-IV data, data from scientific publications, and recommendation data was reformatted into tables and graphs using Microsoft Excel.

Dietary recommendations were taken from the USDA publications of the 2010 DGA and choosemyplate.gov website as well as from publications from the Institute of Medicine (IOM) for vitamin D and calcium intake recommendations. The 2010 DGA are largely based on 2005–2006 NHANES data and the IOM’s Dietary Reference Intakes.

WWEIA information is listed in tables on the USDA website showing the relative percentage and amount of each nutrient consumed. These data are organized by gender, age group, and ethnicity. Energy contributions of each eating occasion (breakfast, lunch, snacks, and dinner) as well as nutrient consumption at each eating occasion are also available on the website.

For children aged two to five (*n* = 861) [10], a parent or caregiver completed the dietary recall information. For children aged six to 11 (*n* = 1132) [10], a parent or caregiver assisted the child with completion of the dietary intake questions.

School lunch data from the SNDA-IV is available online in summary reports. NSLP data from 902 schools, including 316 elementary schools, was collected for the SNDA-IV [12]. Schools across the country were selected for the survey to produce nationally representative data. Data was collected both from School Food Authorities (SFA), or school district groups, and from groupings of SFAs and schools [12]. All public SFAs that participated in the NSLP were considered for participation in the SNDA-IV [12]. SFAs and schools were selected via two sample frames [12]. A sample of SFAs was chosen first [12]. Then, from a second sample of additional SFAs, individual schools were selected for the study based on location, income level, urbanicity, number of students, and SFA size [12]. Two surveys were administered, one to the SFA-only sample and a second survey for the SFA plus schools sample. A total of 595 SFAs were recruited for the study. Five hundred and seventy-eight SFAs completed SFA-level Director Survey [12].

## 3. Children’s Food Intake by Meal

Our results represent information from the primary data sets listed above. Food intake data in children is limited, and the data sets used for our analysis are considered the best information available on eating habits and nutrient intakes of children in the U.S. Self-report data have limitations, but for these surveys, attempts were made to obtain the best information possible. For example, pre-school children are not able to describe their food intake accurately, so parents or caregivers completed the dietary recalls for children aged 2–5 years.

Furthermore, different surveys use different cut-off points for data analysis. Since our information was obtained from published papers and government reports, we were unable to correct for different age ranges. We always point out the age range used in the data set we are quoting.

NHANES surveyed children aged 2–11 years and grouped them into “younger” (ages 2–5) and “older” (ages 6–11) categories. Some reports such as DGA list individuals up to 13 years of age as children and split the children into three groups by age range. Different studies may also include different exclusion criteria, but the goal of these surveys is to obtain a representative sample of American children.

Breakfast: Most children consume at least one food for breakfast, and foods eaten at breakfast provide important micronutrients, especially Vitamin D and calcium [14]. NHANES data shows that breakfast provides 34%–39% of children’s Vitamin D intake and 25% to 28% of their calcium intake [14]. Overall, breakfast contributes to 30%–35% of total daily key vitamin and mineral intake (with “key vitamins and minerals” defined as Vitamin D, B12, calcium, sodium, potassium, and magnesium) [14].

Snacks: Ninety-seven percent of the children surveyed eat a snack, and half of these children eat multiple snacks per day [15]. Snacks contribute to 37% of children’s energy intake [16] but only provide 15%–30% of vital micronutrients [17]. Popular snack choices include desserts and sugar-sweetened drinks [16], and snacks consist of almost 40% of the added sugar in children’s diets [17]. Overall, children aged two to five consume 12 teaspoons of sugar per day, and children aged six to 11 consume about 18 teaspoons of sugar per day [10].

Lunch- SNDA data shows that the average school lunch comes within 10% of the SMI’s standards for its target nutrients [18]. NSLP lunches also generally provided at least one third of the recommended daily amounts of grains, dairy foods, and oil, but they are also high in calories from solid fats and added sugars [13].

According to NHANES data, most children (93%) aged two to five eat lunch, and lunch provides about 25% of their daily energy intake [19]. Lunch accounts for 23% of the consumption of nutrients of concern for these children and also contributes to 20% of their sugar intake [19].

Dinner: Most children consume at least one food item for dinner, an eating occasion that provides 21%–28% of calcium intake and about 20% of Vitamin D, 30% of potassium, and 30% of the dietary fiber consumed by children aged two to 11 [20]. Dinner foods also comprise about 20% of children’s sugar intake [20].

## 4. Children’s Overall Nutrient Intake

Most American children consume snacks, but most of the snacks consumed are energy-rich and nutrient-poor choices, especially considering that children already consume excess energy and insufficient nutrients. Although snacking itself can be an important habit for weight maintenance [21], replacing current snack foods with health-promoting options, especially options naturally abundant in nutrients of concern, would improve children’s diet quality [22].

Certain nutrients of concern, notably calcium and Vitamin D, are sometimes consumed as supplements, however, the Food and Drug Administration, the Academy of Nutrition and Dietetics, and the 2010 DGA recommend consuming foods for adequate nutrition instead of supplements [9]. Based on this recommendation, improving children’s nutrient intake would be best accomplished through changing food consumption habits rather than encouraging supplement usage or reliance. Children already receive most of their calcium and potassium intake from food instead of supplements; more than 97% of calcium consumed by children comes from food alone [23] and almost 100% of children’s potassium intake [23] comes from food.

Yogurt, fruits, and vegetables are naturally rich sources of the 2010 DGA’s nutrients of concern and are also foods that children do not consume sufficiently (Table 1) [9]. Adding one 6 oz serving of yogurt each day would help children move closer to DGA recommendations for almost all of the nutrients of concern [24]; combining yogurt and fruit or yogurt and vegetables for snacks would increase consumption of all of the nutrients of concern. According to the USDA’s National Nutrient Database for Standard Reference, a typical serving of Vitamin D fortified, fruit-flavored, low fat yogurt is about 6 oz and contains 235 mg calcium, 301 mg potassium, 2.2 µg Vitamin D, and 31 g sugar [25]. Adding one daily serving of yogurt as a snack for children ages nine to 11 would provide enough calcium for this group to meet recommended intake levels (Table 2), and a serving of yogurt would increase Vitamin D and potassium consumption for children in all age groups. Children of all age groups do not consume enough of those two nutrients (Table 2).

**Table 1:** NHANES Food Groups: Recommended Intake* versus* Actual Consumption, Comparisons using Recommendation Information from Choosemyplate.gov and Consumption Data from 2009–2010 NHANES data for Children 2–11 Years Old.

Food Group [26]	Recommended Daily Intake [11]	Actual Intake [26]
Dairy foods	2–3 years old (both genders): 2 c 4–8 years old (both genders): 2.5 c 9–13 years old (both genders): 3 c	2–5 years (females): 2.46 c
		2–5 years (males): 2.31 c
		6–11 years (females): 2.03 c
		6–11 years (males): 2.46 c
Fruits	2–3 years old (both genders): 1 c	2–5 years (females): 1.43 c
	4–8 years old (both genders): 1–1.5 c	2–5 years (males): 1.49 c
	9–13 years old (females): 1.5 c	6–11 years (females): 1.20 c
	9–13 years old (males): 1.5 c	6–11 years (males): 1.03 c
Protein foods	2–3 years old (both genders): 2 oz	2–5 years (females): 2.93 oz
	4–8 years old (both genders): 4 oz	2–5 years (males): 3.05 oz
	9–13 years old (females): 5 oz	6–11 years (females): 3.59 oz
	9–13 years old (males): 5 oz	6–11 years (males): 3.97 oz
Vegetables:	2–3 years old (both genders): 1 c	2–5 years (females): 0.69 c
	4–8 years old (both genders): 1.5 c	2–5 years (males): 0.66 c
	9–13 years old (females): 2 c	6–11 years (females): 0.80 c
	9–13 years old (males): 2.5 c	6–11 years (males): 0.78 c
Total Grains: refined and whole grains	2–3 years old (both genders): 3 oz	2–5 years (females): 4.54 oz
	4–8 years old (both genders): 5 oz	2–5 years (males): 4.92 oz
	9–13 years old (females): 5 oz	6–11 years (females): 6.73 oz
	9–13 years old (males): 6 oz	6–11 years (males): 6.75 oz
Whole Grains	2–3 years old (both genders): 1.5 oz	2–5 years (females): 0.61 oz
	3–8 years old (both genders): 2.5 oz	2–5 years (males): 0.79 oz
	9–13 years old (females): 3 oz	6–11 years (females): 0.61 oz
	9–13 years old (males): 3 oz	6–11 years (males): 0.65 oz

**Table 2:** 2010 DGA Nutrients of Concern: Recommended Intake* versus* Actual Consumption, Comparisons using Dietary Reference Intakes or Adequate Intake and 2009–2010 NHANES data for Children 2–11 Years Old.

Nutrient of Concern	Recommended Daily Consumption	Actual Daily Intake (from Food)
Vitamin D	1–3 years old (both genders) [27]: 10 μg	2–5 years old (both genders) [23]: 6.8 μg 6–11 years old (both genders) [23]: 6.1 μg
	4–8 years old (both genders) [27]: 10 μg	
	9–13 years old (both genders) [27]: 10 μg	
Potassium	1–3 years old (both genders) [27]: 3000 mg	2–5 years old (both genders) [23]: 2071 mg 6–11 years old (both genders) [23]: 2172 mg
	4–8 years old (both genders) [27]: 3800 mg	
	9–13 years old (both genders) [27]: 4500 mg	
Calcium	1–3 years old (both genders) [27]: 700 mg	2–5 years old [23]: 1032 mg 6–11 years old (both genders) [23]: 1048 mg
	4–8 years old (both genders) [27]: 1000 mg	
	9–13 years old (both genders) [27]: 1300 mg	
Dietary fiber	1–3 years old (both genders) [27]: 19 g	2–5 years old (females) [10]:11.3 g
	4–8 years old (both genders) [27]: 25 g	2–5 years old (males) [10]:12.1 g
	9–13 years old (females) [27]: 26 g	6–11 years old (females) [10]: 14.5 g
	9–13 years old (males) [27]: 31 g	6–11 years old (males) [10]: 13.6 g

Yogurt manufacturers are already working to decrease the amount of added sugars in yogurt. The amount of added sugars listed by the USDA’s National Nutrient Database for Standard Reference [25], referenced above, does not reflect the amount of added sugars in some yogurt brands. For 6 oz of low fat strawberry yogurt, one national brand contained 21 g of sugar and another national brand contained 24 g, while some yogurt marketed to children can have as few as 18 g of sugar, according to company websites. The amount of sugar in these products is much lower than the amount listed in the USDA’s database. However, not all yogurts on the market have reduced sugar content, and many products still do not meet the IOM’s standard for competitive foods in schools. Although removing all added sugars would likely discourage consumption, especially among children, establishing the IOM’s 23 g of total sugar per 6 oz serving as a recommended maximum amount would allow for a decrease in added sugars without removing them completely.

Finally, the proposed Nutrition Facts labels would decrease the serving size, or Reference Amounts Customarily Consumed, of yogurt from 8 oz to 6 oz [28], a more common size for single-serving yogurt containers [28]. This serving size reduction would allow low fat yogurt to contain 3 g of fat per 6 oz of yogurt instead of 3 g of fat per 8 oz serving. Recent studies suggest that dairy fat and full-fat dairy products confer health benefits that low fat or nonfat dairy products do not [29,30,31]. Choosing a whole milk yogurt instead of a nonfat yogurt may also decrease the need for added sugars to increase palatability, as the fat in yogurt provides flavor and increases satiety. In comparison to a 6 oz serving of fruit-flavored yogurt, 6 oz of plain whole milk yogurt contains a total of 8 g of sugar with amounts of vitamin D (if fortified), calcium, and potassium comparable to that of a low fat, flavored yogurt [25]. Increasing acceptability and availability of whole milk yogurts with low sugar content may be a beneficial way to encourage health-promoting and nutrient-dense snacking among children without raising sugar consumption.

## 5. Limitations of the Review

Using different sources for intake and recommendation information created some limitations to the scope and analysis of this review. As shown in Table 1 and Table 2, data sources for nutritional intake and recommendation information use different age ranges, which complicates comparisons. NHANES, for example, separates data about children into “two to five years old” and “six to 11 years old” categories, while the 2010 DGA has three categories for children: one to three years old, four to eight years old, and nine to 13 years old.

In addition, because data for lunch consumption in this paper comes from NHANES data for children ages five years of age and younger and from school lunch intake data (SNDA-IV) for children ages six and older, and different data are collected for each survey, developing a comprehensive overview of children’s lunch consumption habits was not feasible. For example, the SNDA does not collect data on all of the DGA’s nutrients of concern, including Vitamin D and potassium. This difference may be a cause for concern because it prohibits the development of a complete overview of the nutrient consumption of children while at school and complicates finding and addressing the most prevalent areas of concern in children’s diets.

School lunch data presents an additional complicating factor, because SNDA data reflect the meals that children were offered or served but not consumption data. While children may be offered or served certain foods, data on food consumption is not collected. A recent study on school lunch waste indicated that the measure of food served was not an adequate representation of food consumption at school lunch [32].

Finally, when the data analysis for this review was conducted, only raw data from the 2009–2010 NHANES were available. Analyzed data from the 20092–010 NHANES were not yet available.

## 6. Conclusions

American children aged two to 11 consume extra energy and sugars in their diets but insufficient Vitamin D, calcium, and potassium. One way to address the insufficiencies and excesses of children’s diets would be to change the nutrient density of children’s snacks. Foods high in added sugars and energy currently dominate children’s snack choices. Substituting one serving of low sugar, whole milk yogurt, paired with fruit or vegetables, for current snacks would increase children’s consumption of valuable nutrients without adding excess sugar or energy.
